# Simulation Analysis of Fluid-Structure Interaction of High Velocity Environment Influence on Aircraft Wing Materials under Different Mach Numbers

**DOI:** 10.3390/s18041248

**Published:** 2018-04-18

**Authors:** Lijun Zhang, Changyan Sun

**Affiliations:** 1 National Center for Materials Service Safety, University of Science and Technology Beijing, Beijing 100083, China; 2 School of Chemistry and Biological Engineering, University of Science and Technology Beijing, Beijing 100083, China; cysun@ustb.edu.cn

**Keywords:** aircraft wing, carbon fiber composite material, fluid-structure interaction (FSI), high speed fluid, Mach numbers, stress

## Abstract

Aircraft service process is in a state of the composite load of pressure and temperature for a long period of time, which inevitably affects the inherent characteristics of some components in aircraft accordingly. The flow field of aircraft wing materials under different Mach numbers is simulated by Fluent in order to extract pressure and temperature on the wing in this paper. To determine the effect of coupling stress on the wing’s material and structural properties, the fluid-structure interaction (FSI) method is used in ANSYS-Workbench to calculate the stress that is caused by pressure and temperature. Simulation analysis results show that with the increase of Mach number, the pressure and temperature on the wing’s surface both increase exponentially and thermal stress that is caused by temperature will be the main factor in the coupled stress. When compared with three kinds of materials, titanium alloy, aluminum alloy, and Haynes alloy, carbon fiber composite material has better performance in service at high speed, and natural frequency under coupling pre-stressing will get smaller.

## 1. Introduction

With the rapid development of modern aviation and aerospace industry, velocity and performance of the aircraft have been greatly improved, which makes the aircraft service environment worse. In a higher velocity environment, the aircraft needs to bear greater pressure and temperature [1,2]. For example, when the aircraft speed reaches Mach 7, the aircraft’s local max pressure can exceed 3 MPa, and the local max temperature can reach 2500 °C [3,4], which makes the aircraft wing components more easily damaged. The hypersonic vehicle X-43A is service at about Mach 9.68 [4]. So, it is very important to carry on the research for the non-destructive testing and evaluation (NDT&E) techniques [5,6]. However, in most critical environment, such as high pressure and high temperature, the numerical simulation should be discussed firstly as the basis for the future experiment. Recently, it is a state-of-art attention to study the flow influence on the wing structure that is based on simulation analysis of fluid-structure interaction (FSI).

The FSI problem is a branch of mechanics that is generated by fluid mechanics and solid mechanics, and is also the study of various behaviors by deformable solids in the flow field and the solids deformation affect the flow field as well as the interaction of the two aspects [7,8,9]. To solve such a problem as the FSI system, analytical and semi-analytical methods can be used. But to solve those real engineering problems with complex boundary limitations, which are rather difficult to solve numerical analysis methods with the help of computers, is an effective way; although it can only come up with an approximate result [8,9]. In the aerospace field, the FSI analysis has been widely used. For example, O. Joshi et al. [10] developed a model of surface radiation and thermal interactions between atmospheric gases and the structure of a space vehicle in the phase of atmospheric entry to study the influence on the structure by the heat radiation. S. Tavemiers et al. [11] proposed a conservative domain-decomposition algorithm, in which tight coupling is achieved by employing either Picard’s or Newton’s iterative method. From the above researches, they evinced that heat radiation has a deep influence on the structure at such high temperatures, and the lowering of the temperature distribution due to radiation losses in the solid.

In this paper, the simulation analysis method of FSI is presented to discuss the effect of the temperature and pressure in the flow field on the natural characteristics of different materials for the aircraft wing. The flow field of the aircraft wing structure under different Mach numbers is simulated by Fluent to extract pressure and temperature on the wing; and, the FSI method is used in ANSYS-Workbench to calculate the stress that is caused by pressure and temperature, which will ultimately determine the effect of coupling stress on properties of the wing’s material and structural. Finally four kinds of materials, titanium alloy, aluminum alloy, Haynes alloy, and carbon fiber composite material are compared for their performance in service at high speed. The carbon fiber composite material is proved to gain better performance than other materials.

Section 2 gives the basic theory for the fluid solid coupling equation and the modal analysis equation under pre-stress. Section 3 introduces the numerical simulation of the wing flow field. Numerical analysis of the wing structure is presented in Section 4. In order to show the validity of the simulation model, the discussion of grid convergence study of numerical study is presented in Section 5. Section 6 draws the conclusion of the whole paper.

## 2. Basic Theory

### 2.1. Fluid Solid Coupling Equation

#### 2.1.1. Fluid Equations

Fluid flow is governed by the law of the physical conservation, which basically includes the law of conservation of mass, conservation of momentum, and conservation of energy [12]. If the fluid contains different conditions, the system has to follow the law of components for conservation as well. For general compressible Newtonian fluid, the laws go as the following equations.

Mass conservation equation:

(1) $$\frac{\partial \rho_{f}}{\partial t}+\nabla \cdot (\rho_{f}v)=0$$

Momentum conservation equation:

(2) $$\frac{\partial \rho_{f}v}{\partial t}+\nabla \cdot (\rho_{f}vv-\tau_{f})=f_{f}$$

Energy conservation equation:

(3) $$\frac{\partial (\rho h)}{\partial t}-\frac{\partial p}{\partial t}+\nabla \cdot (\rho_{f}vh)=\nabla \cdot (\lambda \nabla T)+\nabla \cdot (v\cdot t)+v\cdot \rho f_{f}+S_{E}$$

Among above three equations, *t* is the time, *f*_f_ is the volume force vector, *ρ*_f_ is the fluid density, *v* is the fluid velocity vector, *h* is the total enthalpy of the fluid portion, *λ* is the thermal conductivity, *S*_E_ is energy source, *τ*_f_ is the shear stress tensor, and is can be expressed as:

(4) $$\tau_{f}=(-p+\mu \nabla \cdot v)I+2\mu e$$

In Equation (4), p is fluid pressure, μ is the dynamic viscosity, e is the time derivative of the strain tenor, and $e=\frac{1}{2}(\nabla v+\nabla v^{T})$.

#### 2.1.2. Solid Equations

The conservation equation of solid part can be deduced from the Newton’s second law:

(5) $$\rho_{s}{\ddot{d}}_{s}=\nabla \cdot \sigma_{s}+f_{s}$$

In Equation (2), $\rho_{s}$ is Solid density, $\sigma_{s}$ is Cauchy stress tensor, $f_{s}$ is volume of the vector, and ${\ddot{d}}_{s}$ is quality of the local acceleration of the solid domain.

Thermal deformation that is caused by the temperature difference is:

(6) $$f_{T}=\alpha_{T}\cdot \nabla T$$

#### 2.1.3. FSI Equations

In the fluid and solid coupling interface, the conservation of some variables should be as fluid and solid stress (τ), displacement (d), temperature (T), and heat flux (q):

(7) $$\left\{ \begin{array}{l} \tau_{f}\cdot n_{f}=\tau_{s}\cdot n_{s}\\ d_{f}=d_{s}\\ q_{f}=q_{s}\\ T_{f}=T_{s} \end{array}\right.$$

In Equation (7), “f” is for fluid, and “s” is for solid.

### 2.2. Modal Analysis Equation Under Pre-Stress

The modal analysis method aims to identify the modal parameters of the system, which can provide a basis for an analysis and a prediction of the vibration characteristics of the structure. Modal analysis using the finite element method is one of the most commonly and reliable methods. The equation of freedom modal analysis [13] is:

(8) $$([K]-\omega^{2}[M])q=0$$

In Equation (8), [K] is the structural stiffness matrix, [M] is the mass matrix, ω is the vibration frequency, and q is the value of the modal.

When the structure is loaded, it will produce stress and affect the natural frequency. Therefore, in some cases, static structural analysis must be done first, and the equation is:

(9) [K]{x}={F}

Therefore, the modal analysis equation under pre-stress is:

(10) $$(\left[K+K_{s}\right]-\omega^{2}\left[M\right])q=0$$

In Equation (10), $K_{s}$ is the stress stiffness matrix that is caused by pre-stress.

## 3. Numerical Simulation of Wing Flow Field

### 3.1. Condition Description and Model Establishment

**Figure 1:**
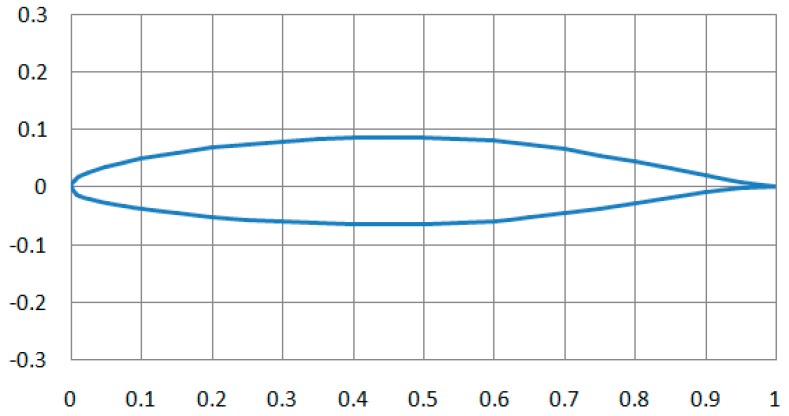
Two-dimensional (2D) model of NACA662-215 airfoil structure.

In this paper, the parameters are used in the simulation analysis of the conventional airfoil (NACA662-215) model, as shown in Figure 1, the chord length *L* = 1 m, the wingspan *D* = 4 m at 4°, the flow rate Mach 0.5, 1, 2, and 4. For the NACA662-215 model, the Gambit software is introduced to model and mesh. For the problem of the flow around a wing of such external disturbance, boundary and solid wall need to be defined away from the wing, and then flow area is constituted and the grid is also made. To keep the outer boundary of the fluid domain with the surrounding environment, the outer boundary should be as far as possible from the wing’s wall. In principle, the farther the boundary is from the wing’ wall, the less influencing it is on the air flow. The combination of semi-cylinder in the Gambit software and cuboids is used in this paper. By Boolean operation, it reduces the three-dimensional wing from the outer boundary, and thus gets the analysis area.

**Figure 2:**
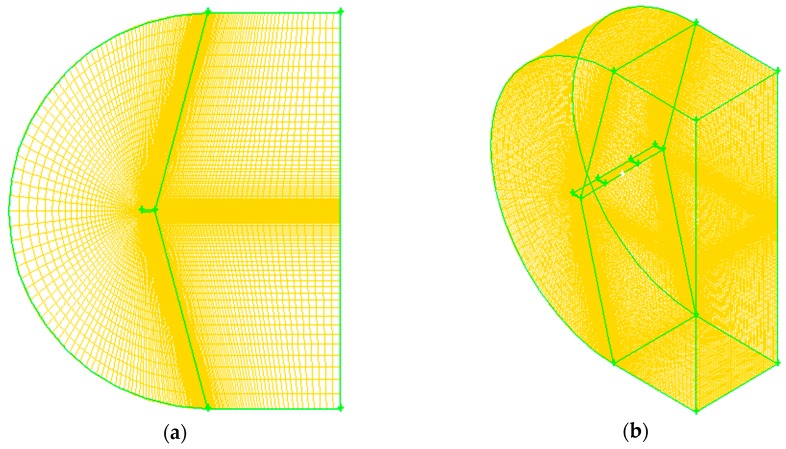
Computation domain grid model. (**a**) 2D model (**b**) 3D model.

When considering the airfoil structure, the dense mesh on the wing surface is needed, and the region away from the wing can be divided relatively loose. Eventually, the model is divided into 655,002 node elements. Then, the peripheral surface is defined as far-field pressure, and the wing surface is defined as a solid wall surface. The computational grid is shown in Figure 2.

### 3.2. Model Simulation and Analysis

The mesh of the wing structure is imported into Fluent. After checking the grid, the implicit solver based on density is selected, because it is more suitable for compressible flow problems and has a faster convergence. Since the airflow around the aircraft body experiences both laminar and turbulent regimes, transition regions and separation points are strongly affected by turbulence modeling in the CFD (computational fluid dynamics) simulations [14]. So, in this paper, the flow field is turbulent, the turbulence model is more appropriate to solve such problems as near wall flow problems, and is good at solving out the boundary layer problem with an adverse pressure gradient. As for a turbulence model, the Spalart-Allmaras model is used because it is relatively simple, which is used to figure out an eddy viscosity transport equation [14,15]. As the Spalart-Allmaras model is one kind of Reynolds-average Navier-Stokes (RANS) model [16], the Reynolds number and the turbulent magnitude are considered accordingly in this paper. 

Therefore, it is often used in solving aerodynamic problems, such as the analysis of the flow field and the flow around the wing of the aircraft. On the control parameters panel, the Roe-FDS flux difference method is selected when Mach 0.5 and AUSM^+^ method when 1, 2, and 4. This method improves the capture efficiency of shock at a high Mach number. The second-order upwind pattern, which is picked when selecting the time difference scheme, is more accurate than the first-order upwind pattern. Then, physical properties of fluid and boundary conditions are selected in accordance with the condition mentioned in the previous section. Finally, steady flow field analysis will be processed with the iteration number of 1000. Then, the monitor of drag and lift coefficient curve is opened and continued 2000 iterative computations.

When three components of velocity, the energy of the residual curve and other coefficient curve tend to be gentle, it indicates that the model has reached a steady state.

**Figure 3:**
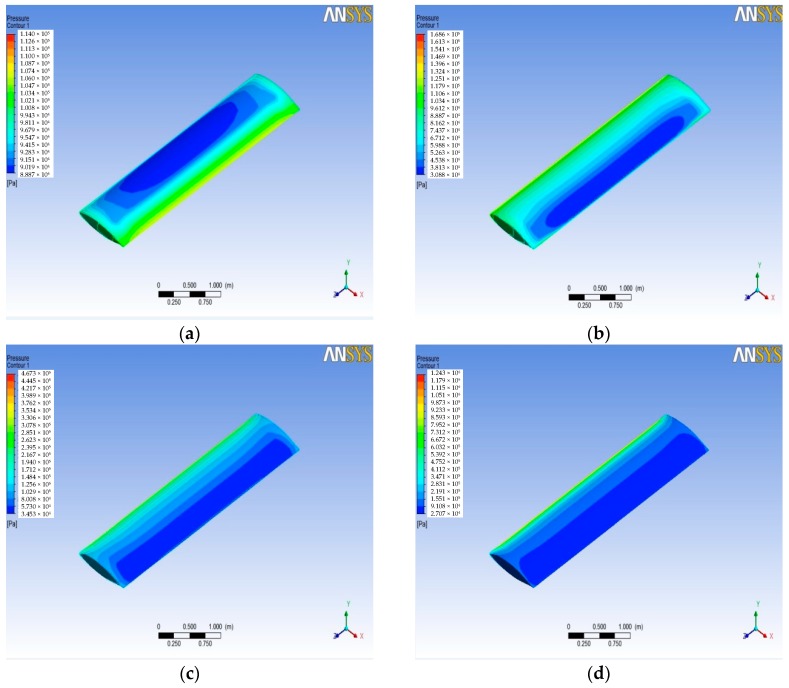
Static pressure distribution on wing surface at different Mach numbers. (**a**) Mach 0.5; (**b**) Mach 1; (**c**) Mach 2; and, (**d**) Mach 4.

**Figure 4:**
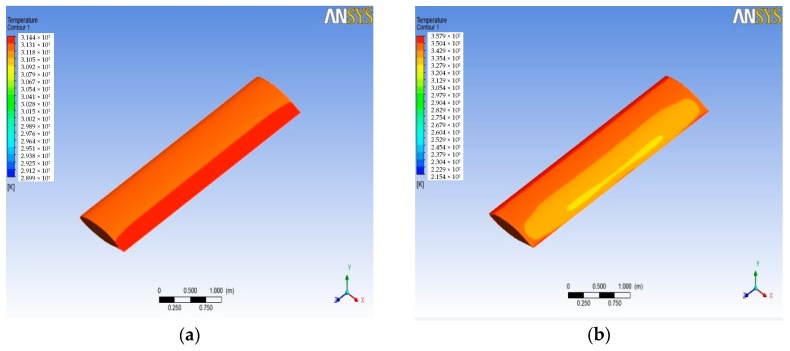

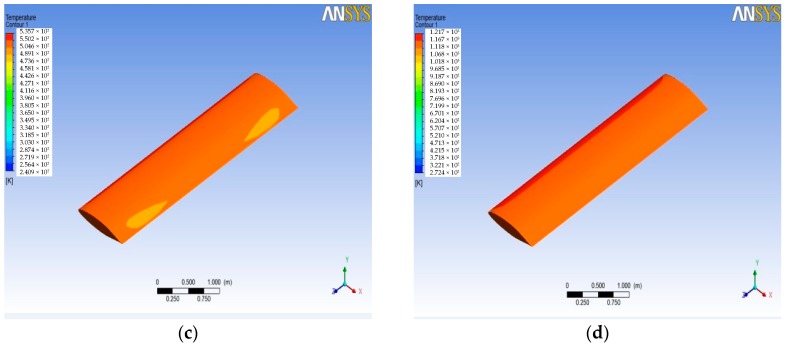
Temperature distribution on wing surface at different Mach numbers. (**a**) Mach 0.5 (**b**) Mach 1 (**c**) Mach 2; and, (**d**) Mach 4.

**Figure 5:**
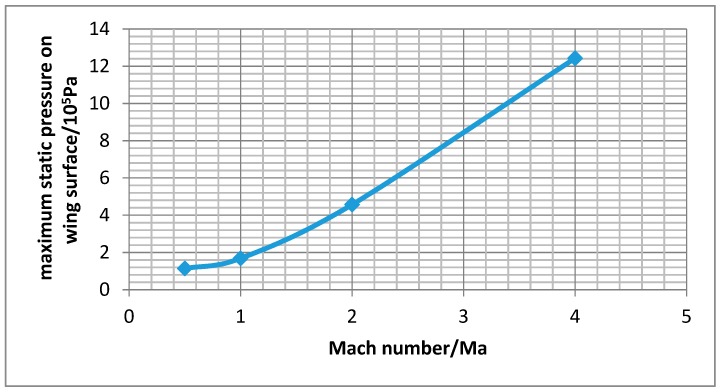
Variation of wing surface to maximum static pressure with the Mach numbers.

**Figure 6:**
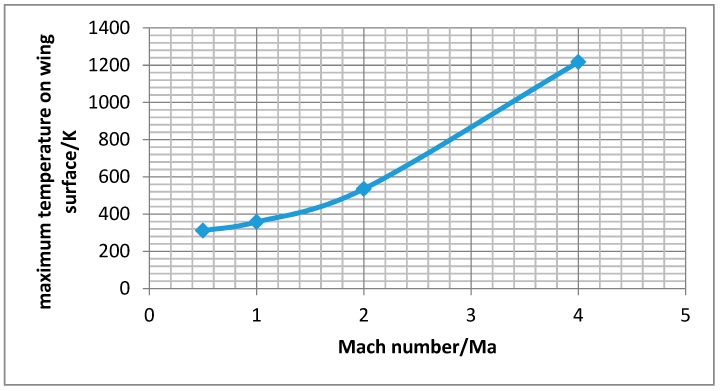
Variation of wing surface to maximum static pressure with the Mach numbers.

After the simulation, the pressure and temperature on the wing surface at different Mach numbers of the wing aircraft can be obtained from the post-processing stage. Figure 3 shows the pressure contours on the wing surface at different Mach numbers. It can be seen that the static pressure gradient on the wing surface passes backward with the increasing Mach number. The maximum static pressure position appeared at the leading edge of the wing surface, and for the surface of the wing trailing edge position, static pressure decreases with the increase of the Mach number. Figure 4 shows the wing surface temperature contours, and the figure shows that when the Mach number is low, the temperature that is generated by the air flow is not high, but when supersonic flight speed increases, the wing surface temperature increases significantly. It will be more than 1200 K, when the flight speed gets Mach 4. The maximum value of wing surface pressure and temperature at different Mach numbers is used to fit a curve to get Figure 5 and Figure 6. It can be seen from Figure 5 and Figure 6 that the change trend of static pressure and temperature in tandem with an increasing Mach number is consistent; obeying the exponential increase, with the Mach number doubling the growing speed of static pressure and the temperature escalating faster and faster.

Here, the Reynolds number *Re* can be calculated as

(11) Re=ρuL/μ

where ρ is the density of the fluid, *u* is the velocity of the fluid with respect to the object, *L* is a characteristic linear dimension, and μ is the dynamic viscosity of the fluid. From Figure 6, the maximum static pressure with the Mach 0.5 is 311.4 K. We can also get ρ 1.135 kg/m^3^, *u* 170 m/s, *L* 1 m, and μ 1.907 × 10^−5^. Then, *Re* is 1.13 × 10^7^ as a high Reynolds number (*Re* > 4000). So, the turbulence model is also proved in this simulation. 

In general, for the high Reynolds number model, the turbulent magnitude y+ needs to generally satisfy that it is close to 30; for the low Reynolds number model, it needs to be close to 1. Therefore, when estimating the first layer of the grid, y+ is estimated using 30 since the turbulence model is selected in this paper.

## 4. Numerical Analysis of Wing Structure

### 4.1. Establishment of Finite Element Model Structure

The internal structure of the wing is very complex, which includes the skin, rivets, reinforcing ribs, spars, and others [17]. In order to apply finite element analysis, it is simply assumed that the material inside the wing is solid satisfying the hypothesis of continuity, homogeneity, and isotropy.

**Figure 7:**
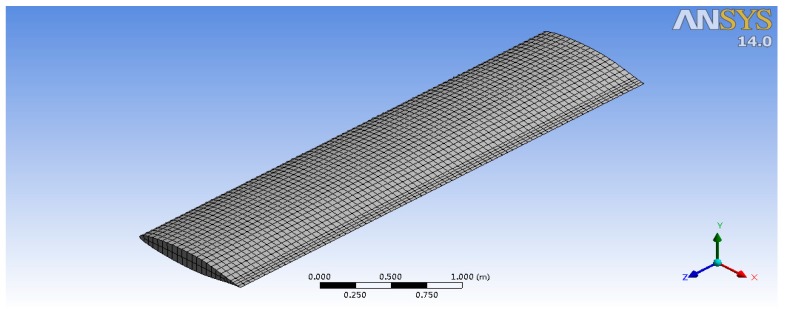
Wing structural finite element model.

First, the structural model of the wing is generated. Then, the model is imported into an ANSYS-Workbench and meshed. The structure mesh model of the wing is shown in Figure 7. Titanium alloy is chosen for the coupled simulation in this paper as the wing material to analyze the stress that is caused by pressure and temperature that has an influence on the wing’s structure at different Mach numbers. Then titanium alloy, aluminum alloy, Haynes alloy, and carbon fiber composite material, four kinds of different materials are used to analyze the influence of the coupling stress on the material at the same high flow velocity (Mach 4). The material properties of titanium alloy and the aluminum alloy are the default variables in ANSYS-Workbench, and the material properties of Haynes alloy and carbon fiber composite material are from [18,19], respectively. Four kinds of material name and attribute are shown in Table 1.

### 4.2. Stress Analysis

**Figure 8:**
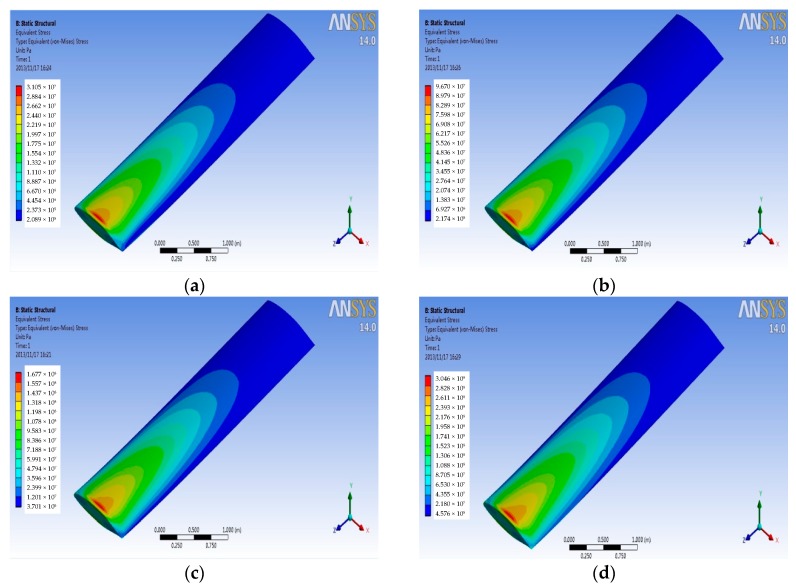
Pressure stress contours at different Mach numbers. (**a**) Mach 0.5 (**b**) Mach 1 (**c**) Mach 2; and, (**d**) Mach 4.

**Figure 9:**
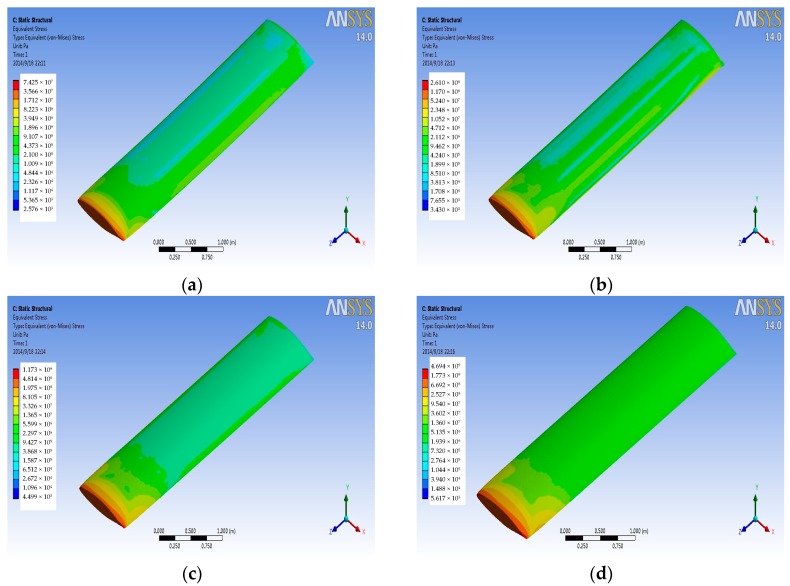
Thermal stress contours at different Mach numbers. (**a**) Mach 0.5 (**b**) Mach 1 (**c**) Mach 2; and, (**d**) Mach 4.

**Figure 10:**
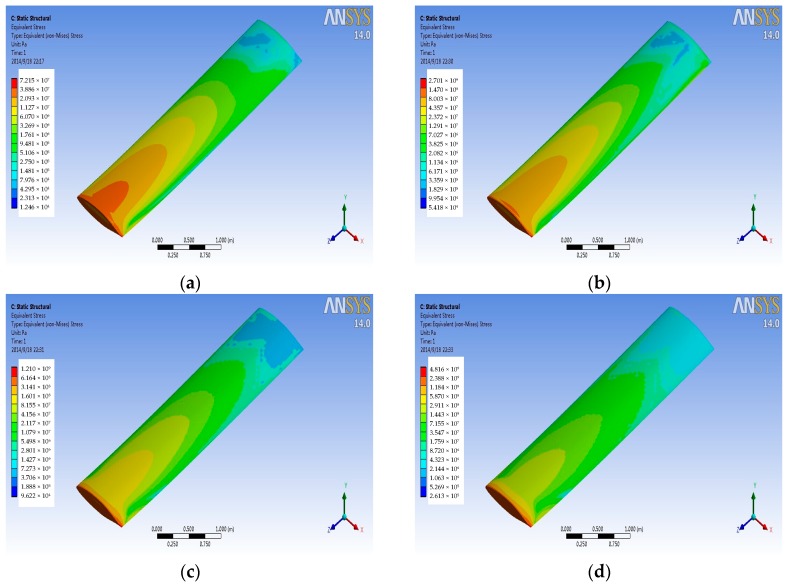
Coupling stress contours at different Mach numbers. (**a**) Mach 0.5 (**b**) Mach 1 (**c**) Mach 2; and, (**d**) Mach 4.

The finite element of airfoil structure is fixed constraints for left face, assuming that the left wing is connected with the aircraft fuselage. The pressure and temperature data on the wing surface at different Mach numbers from the FSI simulation are transferred to the titanium alloy structural finite element model to get the stress distribution on the titanium alloy wing structure in a variety of conditions. Figure 8 and Figure 9 are the stress contours for the wing structure of the titanium alloy, with the former imported only pressure data and the later imported only temperature data. As can be seen from the figure, stress distribution and the maximum stress position for the wing structure are basically the same for different Mach numbers, but with the increase of the Mach number, the magnitude of stress has changed. Figure 10 is the contour of coupling stress that is caused by pressure and temperature for the wing structure of titanium alloy at different Mach numbers. From Figure 9, the maximum stress is at the trailing edge of the wing. Therefore, it can be determined in the course of service that whether the actual stress can meet the limiting stress under stress magnitude of this point. Through a comparison of last three figures (Figure 8, Figure 9 and Figure 10), it can be drawn in the same Mach number, and the thermal stress is larger than the stress that is caused by the pressure for titanium alloy material. Furthermore, with the increase of Mach number, the proportion of the thermal stress is growing, and when the aircraft reaches Mach 4, the thermal stress will reach 10 times of the pressure stress.

**Figure 11:**
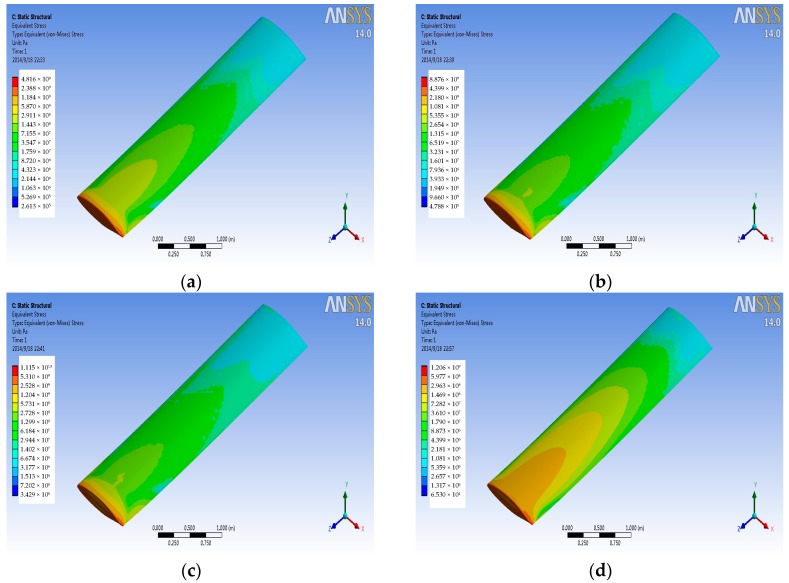
Coupling stress contours of different material at Mach 4. (**a**) Titanium alloy (**b**) Aluminum alloy (**c**) Haynes alloy; and, (**d**) Carbon fiber composite material.

Pressure and temperature data of wing surface from the flow field analysis at Mach 4 are imported into four kinds of finite element models of material wing structure. The coupling stress distribution under the high flow rate is obtained, as shown in Figure 11. From Figure 11, the coupling stress distribution of titanium alloy, aluminum alloy, and Haynes alloy is basically the same; but, titanium alloy suffered the least coupling stress among these three kinds of materials. So, titanium alloy has a better performance under the service of the high flow rate. For the stress contour of carbon fiber composite material, the stress distribution and the maximum stress value are basically same as Figure 10, in which the simulation result of titanium alloy at Mach 2. Therefore, carbon fiber composite material has a better performance than titanium alloy material. Above all, carbon fiber composite material has the best performance among the four kinds of materials under the service of the high pressure and high temperature.

### 4.3. Modal Analysis

**Figure 12:**
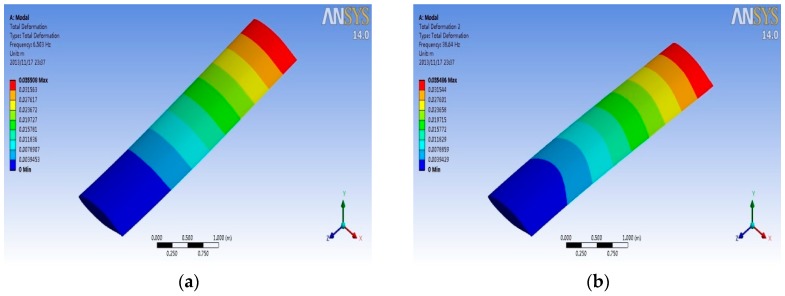

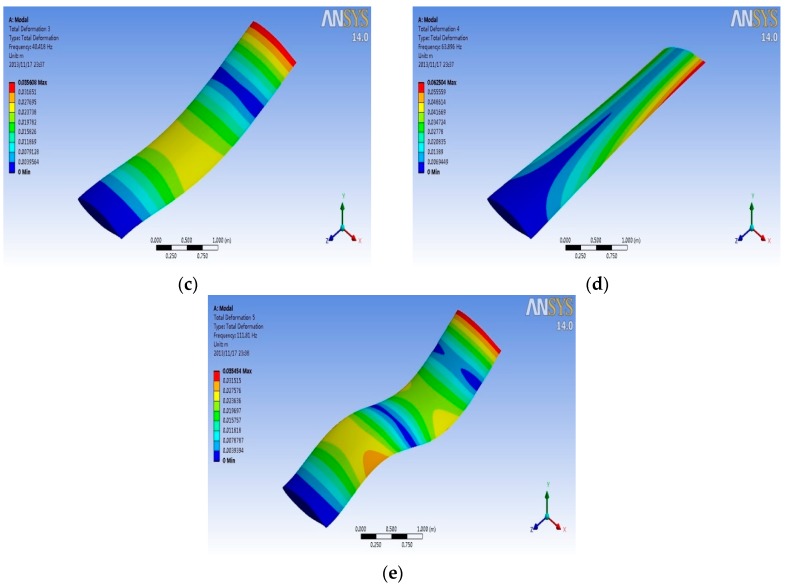
Patterns of modal vibration. (**a**) the first-order modal (**b**) the second-order modal (**c**) the third-order modal (**d**) the forth-order modal; and, (**e**) the fifth-order modal.

In order to study the effect of the coupling stress on the inherent characteristics of the wing structure at different Mach numbers, titanium alloy material is used for the wing structure to have the modal analysis in five different conditions. Condition 1: no pre-stress; Condition 2 to Condition 5: the coupling stress as a pre-stress result at Mach 0.5, 1, 2, and 4, respectively. Then, calculate the first five-order natural frequencies. Natural frequencies and vibration sizes in different conditions are shown as Table 2 and Figure 12 (only one vibration type is listed for each modal due to the slight difference for every condition). As can be seen from Table 2, the inherent frequencies of the wing structure in the presence of pre-stress are lower than the natural frequencies of the wing structure without pre-stress, and the larger the coupling pre-stress, the lower the frequency.

## 5. Discussion of Grid Convergence Study of Numerical Study

Normally, a grid convergence study is a must in any numerical study. In the following, we will focus on the influence of boundary conditions on the convergence of the mesh.

In this paper, the so-called numerical method of fluid computation is to solve the turbulence model under the premise of satisfying the basic conservation law. Only under certain initial conditions and boundary conditions can we obtain a unique solution. Therefore, an important issue for the numerical solution of fluids is how to give boundary conditions. If the boundary conditions are not properly handled, then it may have a great impact on the results of the numerical simulation, and it may result in the results not being converged in the calculation process. This paper mainly uses the far field boundary and the wall boundary in setting the boundary conditions.

(1) Far field boundary conditions.

This boundary condition represents a free flow with an infinite distance from the computing core region. When compared with other boundary conditions, the advantage of the far-field boundary condition is that there is no need to set too many parameters, and only the Mach number of the free flow is required. Other boundary conditions may choose default values. But, its limitation is that it can only be calculated with the ideal gas. For the concept of “distance infinity”, different issues should be generally considered. For example, in the airfoil simulation, the pressure far-field boundary is generally about 20 times the chord length of the model.

The pressure far-field boundary condition is not a boundary condition that is directly described by a constant. It uses certain simulation methods to import Riemann constants indirectly. In the general problem, there are usually two constants, they are defined as waves into and out of the element, which can be expressed as:

(12) $$R_{\infty }=v_{n\infty }-\frac{2c_{\infty }}{\gamma -1}$$

(13) $$R_{i}=v_{ni}-\frac{2c_{i}}{\gamma -1}$$

where vn is the normal speed, *c* is the sound speed, γ is the specific heat ratio. The subscript ∞ indicates the far field, and the subscript i indicates the inside of the calculation domain.

(2) Wall boundary conditions.

This boundary condition indicates the location of the boundary of the basin with the surface of the structure. When performing numerical calculations, the following no-slip, adiabatic wall, and normal zero pressure gradient conditions are used at the wall surface:

(14) $$\left\{ \begin{array}{l} u=0\\ v=0\\ w=0\\ \partial T/\partial \vec{n}=0\\ \partial \rho /\partial \vec{n}=0 \end{array}\right.$$

For an ideal gas, its expression is p=ρRT. If this condition is established, then the normal gradient of p and T is equal to zero, so ρ must satisfy this condition, i.e., $\partial \rho /\partial \vec{n}=0$.

## 6. Conclusions

With the FSI simulation analysis method, this paper conducts flow field analysis of working wing structure under different Mach numbers and studies the influence of temperature and pressure that is caused by flight wing in the flow field on its material and structure. The results show that:

(1) With the increase of Mach number, the pressure and temperature in service increase exponentially. From the simulation experiment, when the speed of the aircraft reach Mach 4, the wing static pressure on a wing surface can reach 1.2 MPa and the temperature will be above 1200 K. So, it is very important for aircraft servicing in the extreme environment to use a high property material.
(2) Pressure stress and thermal stress are produced by fluid on the wing structure. With the increase of the Mach number, the proportion of thermal stress will increase as well, and eventually it will become the main source of the coupling stress. So, for the high velocity environment, the ability of resisting a high temperature should be used as the main index of the wing of an aircraft material.
(3) When compared with titanium alloy, aluminum alloy, and Haynes alloy, the carbon fiber composite material has better performance in service at high speed. The natural frequency under coupling pre-stressing caused by pressure and temperature will get smaller. It can provide the theory basis for selection of aircraft material.
(4) In this paper, the far field boundary and the wall boundary are used in setting boundary conditions. In order to get the convergence for the grid, boundary conditions should be carefully selected according the guidelines.

## Figures and Tables

**Table 1:** Information of aircraft wing material.

	Name	Titanium Alloy	Aluminum Alloy	Haynes Alloy	Carbon Fiber Composite Material
Property					
Elastic modulus E (Pa)		$9.6\times 10^{10}$	$7.1\times 10^{10}$	$1.54\times 10^{11}$	$1.35\times 10^{11}$
Poisson’s ratio v		0.36	0.33	0.3	0.33
Density ρ $\left(\mathrm{kg}/m^{3}\right)$		4620	2270	9130	1610
Shear modulus G (Pa)		$3.53\times 10^{10}$	$2.67\times 10^{10}$	$5.92\times 10^{10}$	$5.1\times 10^{10}$
Thermal conductivity λ (W/(m⋅K))		21.9	170	26.7	6.5
Thermal Conductivity α $\left(K^{-1}\right)$		$9.4\times 10^{-6}$	$2.3\times 10^{-5}$	$1.18\times 10^{-5}$	$1.5\times 10^{-6}$

**Table 2:** Modal frequencies in different conditions.

Modal	Condition 1	Condition 2	Condition 3	Condition 4	Condition 5
1	6.503	5.8804	5.8848	5.8781	5.8694
2	38.64	34.966	34.924	34.923	34.923
3	40.418	36.663	36.63	36.62	36.597
4	63.896	56.41	56.359	56.246	56
5	111.81	101.69	101.28	101.27	101.23
